# Novel Dopamine Transporter Inhibitor, CE-123, Ameliorates Spatial Memory Deficits Induced by Maternal Separation in Adolescent Rats: Impact of Sex

**DOI:** 10.3390/ijms231810718

**Published:** 2022-09-14

**Authors:** Pawel Grochecki, Irena Smaga, Paulina Surowka, Marta Marszalek-Grabska, Predrag Kalaba, Vladimir Dragacevic, Patrycja Kotlinska, Malgorzata Filip, Gert Lubec, Jolanta H. Kotlinska

**Affiliations:** 1 Department of Pharmacology and Pharmacodynamics, Medical University, Chodzki 4A, 20-093 Lublin, Poland; 2 Department of Drug Addiction Pharmacology, Maj Institute of Pharmacology, Polish Academy of Sciences, Smetna 12, 31-343 Krakow, Poland; 3 Department of Experimental and Clinical Pharmacology, Medical University, Jaczewskiego 8B, 20-090 Lublin, Poland; 4 Department of Pharmaceutical Chemistry, Faculty of Life Sciences, University of Vienna, 1010 Vienna, Austria; 5 Paracelsus Private Medical University, 5020 Salzburg, Austria; 6 Department of Neurology, SP ZOZ MSWiA Hospital, 35-111 Rzeszow, Poland

**Keywords:** dopamine, CE-123, learning and memory, rat maternal separation model, DAT

## Abstract

Maternal separation (MS) is a key contributor to neurodevelopmental disorders, including learning disabilities. To test the hypothesis that dopamine signaling is a major factor in this, an atypical new dopamine transporter (DAT) inhibitor, CE-123, was assessed for its potential to counteract the MS-induced spatial learning and memory deficit in male and female rats. Hence, neonatal rats (postnatal day (PND)1 to 21) were exposed to MS (180 min/day). Next, the acquisition of spatial learning and memory (Barnes maze task) and the expression of dopamine D1 receptor, dopamine transporter (DAT), and the neuronal GTPase, RIT2, which binds DAT in the vehicle-treated rats were evaluated in the prefrontal cortex and hippocampus in the adolescent animals. The results show that MS impairs the acquisition of spatial learning and memory in rats, with a more severe effect in females. Moreover, the MS induced upregulation of DAT and dopamine D1 receptors expression in the prefrontal cortex and hippocampus in adolescent rats. Regarding RIT2, the expression was decreased in the hippocampus for both the males and females, however, in the prefrontal cortex, reduction was found only in the females, suggesting that there are region-specific differences in DAT endocytic trafficking. CE-123 ameliorated the behavioral deficits associated with MS. Furthermore, it decreased the MS-induced upregulation of D1 receptor expression level in the hippocampus. These effects were more noted in females. Overall, CE-123, an atypical DAT inhibitor, is able to restore cognitive impairment and dopamine signaling in adolescent rats exposed to MS—with more evident effect in females than males.

## 1. Introduction

Early life experiences are thought to play a key role in brain function and behavior [1]. Experimental and clinical studies have shown that the immaturity and plasticity of the central nervous system (CNS) during childhood make it particularly sensitive to stress at a young age, which may cause significant changes in brain structure and function [1]. In humans, exposure to early life stress has been linked to a variety of affective and addictive disorders in later life [2,3,4,5], and to cognitive disorders such as learning and memory deficits [6,7].

The maternal separation (MS) protocol is a well-known animal model that resembles the stress of early life negative experiences and is considered an analog of childhood abuse or mistreatment [8]. In this animal model, the forced absence of the dam produces alterations in neuroendocrine, cognitive, and behavioral functioning, as well as plastic changes in the offspring that persist into adulthood [9,10,11,12]. Additionally, several published studies have reported that MS modifies the activity of different neurotransmitters, including dopamine [1,13], which could be responsible for the cognitive/spatial memory deficits observed in adolescent animals exposed to the MS model [14].

The hippocampus and prefrontal cortex have been known to play crucial roles in spatial learning and memory processes [15,16]. The hippocampus is particularly relevant for the maintenance of memory [17]. Successful spatial learning requires that hippocampal place cells and location-encoding pyramidal neurons [18] display consistent and stable patterns of neural activity, a process that can be enhanced by selective attention to spatial cues and by the presence of dopamine agonists [19,20]. Conversely, dopamine receptor blockade attenuates the ability of spatial attention to stabilize the firing pattern of hippocampal place cells [20]. Additionally, rats with mesohippocampal dopamine lesions are significantly impaired in the retention of spatial information [21]. According to previous research, early life stress produces long-term prefrontal cortex and hippocampal alteration [22]. 

Evidence supports the notion that dopamine signaling and its receptors have broad influence over learning, memory, and synaptic plasticity [23,24,25,26]. Dopamine D1-like receptors in the hippocampus are especially noted for their critical role in spatial memory [27,28], and activating these receptors could enhance spatial learning and memory [25]. More recently, it has been reported that D1/D5 receptor signaling is associated with long-term memory consolidation [29] and working memory [30]. Thus, dopamine D1 receptor activation in the hippocampus regions can enhance memory and modulate memory consolidation or retrieval [31,32,33]. 

Extracellular dopamine levels are tightly restricted by the presynaptic dopamine transporter (DAT). Following evoked dopamine release, DAT takes up released dopamine back into dopaminergic presynaptic terminals, thereby limiting the spatial and temporal dopamine signal [34]. DAT not only limits extracellular dopamine levels but is also critical to maintain homeostatic dopamine levels and synaptic dopaminergic tone across multiple species [35]. DAT’s pivotal role in regulating dopaminergic signaling in cognitive functions is best illustrated by observing the consequences that follow from either pharmacological DAT inhibition or genetic DAT perturbation. For example, DAT mutant (knockout, KO) animals show deficits in spatial learning abilities [36]. It was found that DAT-KO rats are able to learn the behavioral task, but the level of task performance did not reach that of the wild-type group in spatial memory tasks (i.e., the 8-arm radial maze test). The behavioral tactics used by animals during training significantly differ in mutants. The behavioral tactics used by DAT-KO rats involved perseverations and resulted in worse task fulfillment, in comparison to wild-type controls [36,37]. 

Currently, atypical DAT blockers such as modafinil and methylphenidate are used as cognitive enhancers in aged and cognitively impaired subjects [38,39,40]. They promote central DA signaling, but due to the low specificity of these drugs to other monoamine transmitters, cause numerous additional CNS effects [41,42,43]. Consequently, novel modafinil analogues with improved pharmacological properties with a higher selectivity towards the DAT than norepinephrine and serotonin transporters have been synthesized [44,45]. 

CE-123 is a novel DAT inhibitor that targets the DAT with high specificity [46,47]. Previous research has shown that CE-123 improves memory acquisition and memory retrieval in the spatial memory task [47,48] at doses that did not change the locomotor activity of the animals. Moreover, it shows a minor impact on the reward-related dopaminergic area [49].

Recent reports indicate that DAT function and surface expression are not static but are dynamically modulated by membrane trafficking. It has been demonstrated that the RIT2 protein is a neuron-specific small guanosine triphosphatase (GTPase), a member of the Ras superfamily that has been shown to be expressed in a subset of neurons, including retinal ganglion cells (RGCs) and selected neurons in the brain [50,51]. RIT2 has been indicated to have important roles in neuronal differentiation and function. Furthermore, RIT2 has an important role in intracellular signaling and interacts with DAT in lipid raft microdomains: this interaction is required for protein kinase C (PKC)-regulated DAT trafficking in the nervous system [51]. Additionally, published data described the region- and sex-dependent differences in DAT trafficking regulation by RIT2 [52].

The aim of the present study was to determine whether MS (PND1-21, 180 min/day) affects the development of spatial memory (Barnes maze task) in rats and whether this effect is sex dependent. Using ELISA assays, we evaluated MS-induced changes in the DAT, dopamine D1 receptor expression and RIT2 level in the prefrontal cortex and hippocampus. Finally, we determined whether CE-123, a novel atypical DAT inhibitor, has an impact on the learning acquisition impaired by MS in adolescent rats.

## 2. Results

### 2.1. Effect of CE-123 on Acquisition of Spatial Learning of the Barnes Maze in Young Male and Female Rats with Prior MS

**Figure 1:**
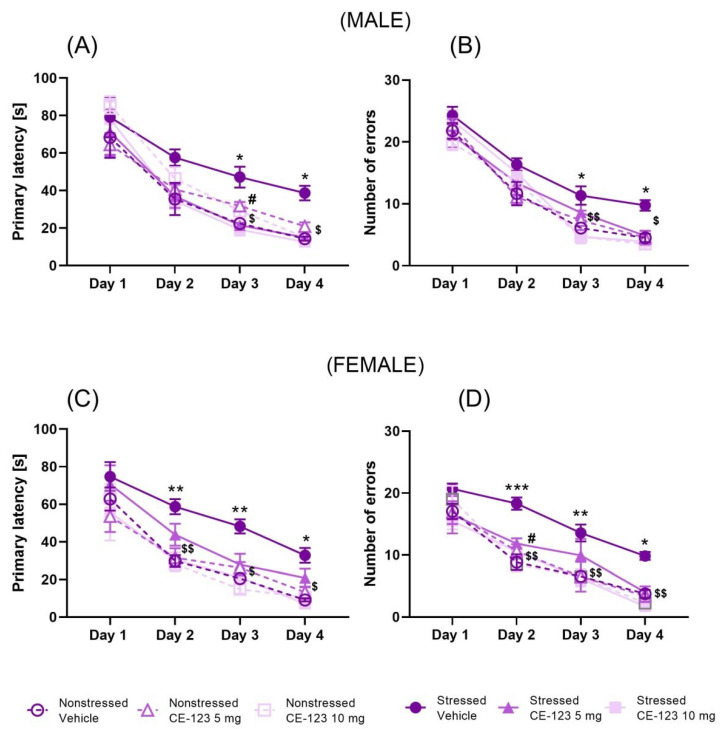
Effect of CE-123 (5 mg/kg and 10 mg/kg) or vehicle given each day before the acquisition session on the primary latency for males (**A**) and females (**C**) and number of errors for males (**B**) and females (**D**) committed during 4 days of the acquisition of spatial learning of the Barnes maze task in adolescent (PND30-33) rats. Wistar rats were subjected to MS for 180 min during PND1-21. Data represent mean ± SEM (N = 10 rats/group). * *p* < 0.05, ** *p* < 0.01, *** *p* < 0.001 Stressed/Vehicle vs. Nonstressed/Vehicle; # *p* < 0.05 Stressed/CE-123 5 mg/kg vs. Stressed/Vehicle; ^$^ *p* < 0.05, ^$$^ *p* < 0.01; Stressed/CE-123 10 mg/kg vs. Stressed/Vehicle; PND—postnatal day.

Male: We examined the deleterious effect of MS on spatial memory acquisition in adolescent male rats. Acquisition of spatial learning in the training phase was evaluated by the decrease in latency time to reach the escape box for four days (PND30–33). A two-way ANOVA with repeated measures showed the significant effect of day of acquisition learning (F(3,216) = 101.0; *p* < 0.001) and the group of rat effect (F(5,216) = 7.157; *p* < 0.001), but no interaction between these factors (F(15,216) = 0.9264; *p* > 0.05) in young male rats (PND30–33). Moreover, the post-hoc Tukey’s test demonstrated that maternal separation increased primary latency at days 3rd and 4th. Moreover, pre-treatment with CE-123 at the dose of 5 and 10 mg/kg decreased the primary latency on the 3rd (*p* < 0.05) day of the acquisition learning trial, while the day 4th only dose 10 mg/kg decreased the primary latency in comparison to the MS group (*p* < 0.05) (Figure 1A). In the number of primary errors committed by the rats, a two-way ANOVA with repeated measures indicated the significant effect of day of acquisition learning (F (3,216) = 214.8; *p* < 0.001) and group of rats (F(5,216) = 7.69; *p* < 0.001), but no interaction between these two factors in acquisition learning (F(15,216) = 1.177; *p* > 0.05) in young male rats (PND30–33). The post-hoc Tukey’s test demonstrated significant increase in number of errors in the MS group at days 3rd and 4th. Furthermore, pre-treatment with CE-123 at the dose of 10 mg/kg significantly decreased the number of primary errors on the 3rd (*p* < 0.01) and 4th day (*p* < 0.05) of the acquisition learning trial (Figure 1B). Thus, CE-123 decreased the deleterious effect of MS on the acquisition of spatial memory in adolescent male rats.

Female: We studied the deleterious effect of MS on spatial memory acquisition in adolescent female rats. A two-way ANOVA with repeated measures showed the significant effect of day of acquisition learning (F(3,216) = 78.78; *p* < 0.001) and group of rat effect (F(5,216) = 12.17; *p* < 0.001), but no interaction between these factors (F(15,216) = 0.5267; *p* > 0.05) in young female rats (PND30–33). Moreover, the post-hoc Tukey’s test demonstrated that MS increased the primary latency in the Barnes maze task acquisition at days 2, 3 (*p* < 0.01), and 4 (*p* < 0.05). In addition, pre-treatment with CE-123 at the dose of 10 mg/kg decreased the primary latency on the 2nd (*p* < 0.01), 3rd, and 4th day (*p* < 0.05) of the acquisition learning trial (Figure 1C). In the number of primary errors committed by the rats, a two-way ANOVA with repeated measures indicated significant effect of day of acquisition learning (F(3,216) = 94.22; *p* < 0.001) and group of rats (F(5,216) = 13.82; *p* < 0.001), but no interaction between these two factors in acquisition learning (F(15,216) = 0.8899; *p* > 0.05) in young female rats (PND30–33). The post-hoc Tukey’s test demonstrated that MS increased the number of errors at days 2 (*p* < 0.001), 3 (*p* < 0.01), and 4 (*p* < 0.05). Moreover, pre-treatment with CE-123 at the dose of 5 mg/kg significantly decreased the number of primary errors on the 2nd day (*p* < 0.05) of the acquisition learning trial (Figure 1D). In addition, the post-hoc Tukey’s test demonstrated that pre-treatment with CE-123 at the dose of 10 mg/kg significantly decreased the number of primary errors on the 2nd (*p* < 0.01), 3rd (*p* < 0.01) and 4th days (*p* < 0.05) of the acquisition learning trial (Figure 1D). Overall, CE-123 strongly ameliorated the MS-induced deficits on the acquisition of spatial memory in adolescent female rats.

### 2.2. Effect of CE-123 Given during Acquisition Session on Spatial Memory Retention in the Probe-Trial of the Barnes Maze in Young Male and Female Rats with Prior MS

**Figure 2:**
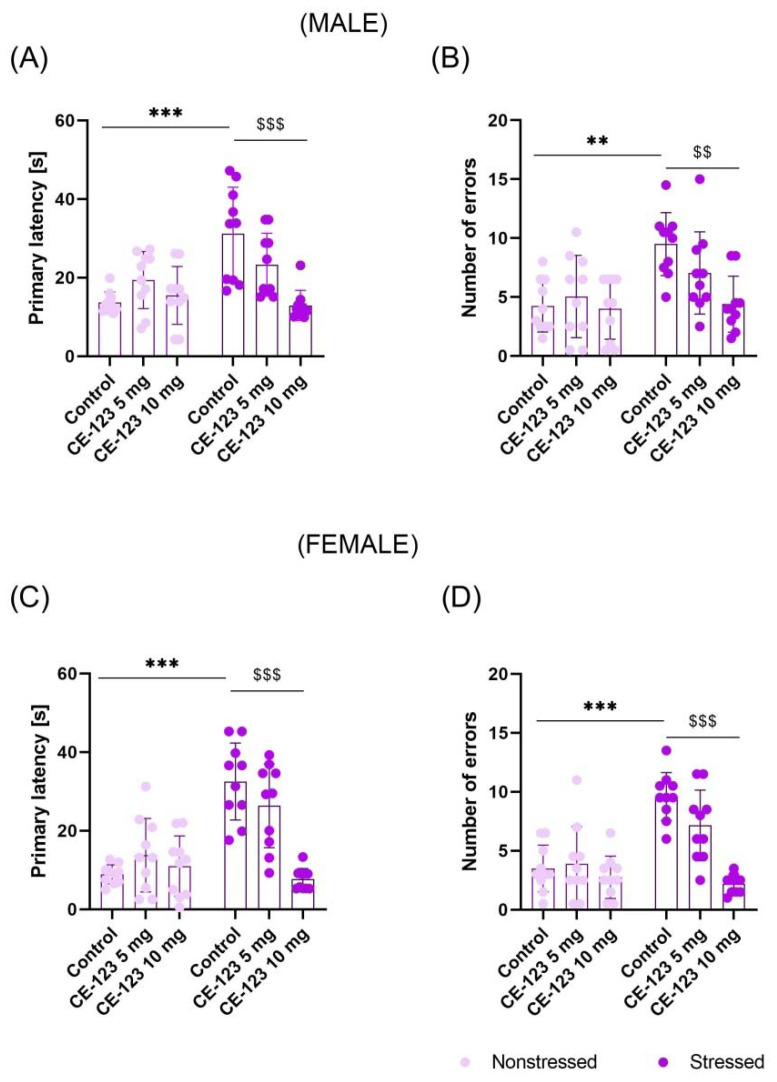
Impact of CE-123 (5 mg/kg and 10 mg/kg) or vehicle given prior to each acquisition session on the primary latency for males (**A**) and females (**C**) and number of errors for males (**B**) and females (**D**) in the probe-trial of the Barnes maze task in adolescent (PND34) rats. Wistar rats were subjected to MS for 180 min during PND1-21. Data represent mean ± SEM (N = 10 rats/group). ** *p* < 0.01, *** *p* < 0.001 Stressed/Vehicle vs. Nonstressed/Vehicle; ^$$^ *p* < 0.01, ^$$$^ *p* < 0.001; Stressed/CE-123 10 mg/kg vs. Stressed/Vehicle; PND—postnatal day.

Male: The probe-trial was conducted 24 h after the last acquisition session. Two-way ANOVA with repeated measures indicated the significant effect of MS (F(1,54) = 10.65; *p* < 0.01), CE-123 administration (F(2,54) = 7.218; *p* < 0.01) and interaction between these factors (F(2,54) = 9.436; *p* < 0.001). Tukey’s test revealed that MS increased primary latency as compared to nonstressed rats (*p* < 0.001) and CE-123 given each day before acquisition sessions at the dose of 10 mg/kg decreased this effect (*p* < 0.001) (Figure 2A). Additionally, these factors (MS (F(1,54) = 12.01; *p* < 0.01); CE-123 (F(2,54) = 4.622; *p* < 0.05); interaction (F(2,54) = 3.761; *p* < 0.05)) had significant effects on the number of errors committed by rats during the probe-trial. Post-hoc test showed that MS increased the number of errors (*p* < 0.01) when compared to nonstressed rats. CE-123 given before every acquisition session at the dose of 10 mg/kg counteracted this MS-induced effect (*p* < 0.01) (Figure 2B), suggesting that CE-123 increased memory retention in MS male rats.

Female: The probe-trial was conducted 24 h after the last acquisition session. Two-way ANOVA with repeated measures indicated the significant effect of MS (F(1,54) = 29.35; *p* < 0.001), CE-123 administration (F(2,54) = 13.30; *p* < 0.001), and interaction between these factors (F(2,54) = 14.93; *p* < 0.001). Tukey’s test revealed that MS increased primary latency in comparison to nonstressed rats (*p* < 0.001) and CE-123 given before each acquisition session at the dose of 10 mg/kg counteracted this effect (*p* < 0.001) (Figure 2C). Additionally, these factors (MS (F(1,54) = 25.16; *p* < 0.001); CE-123 (F(2,54) = 17.52; *p* < 0.001); interaction (F(2,54) = 10.85; *p* < 0.01)) had significant effects on the number of errors committed by rats during the probe-trial. Post-hoc test showed that MS increased the number of errors (*p* < 0.001) as compared to the nonstressed control. However, CE-123 given before every acquisition session at the dose of 10 mg/kg ameliorated this MS-induced effect (*p* < 0.001) (Figure 2D), suggesting its beneficial effect on memory retention in female rats.

### 2.3. The Influence of Early MS on Dopamine Transporter (DAT), GTP-Binding Protein RIT2, and D1 Receptor Expression in the Prefrontal Cortex and Hippocampus in Adolescent Male and Female Rats

#### 2.3.1. Prefrontal Cortex

**Figure 3:**
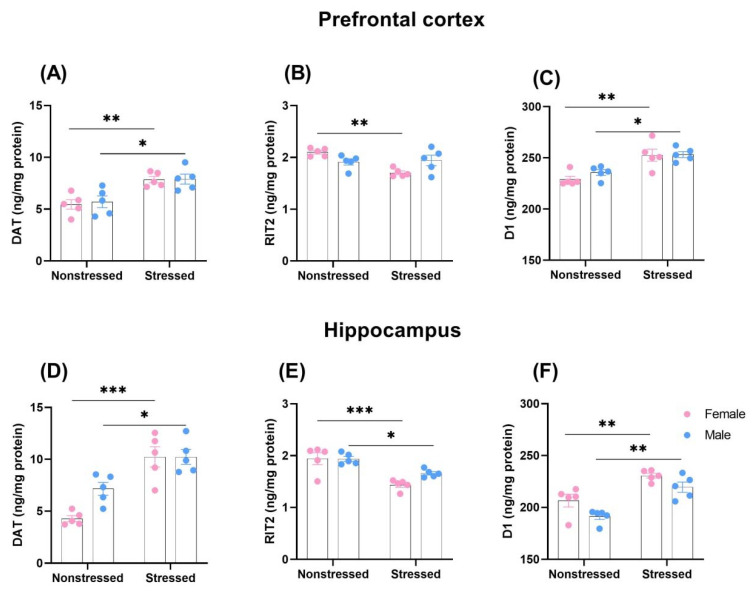
Effect of MS on dopamine transporter (DAT; (**A**,**D**)), GTP-binding protein RIT2 (**B**,**E**), and D1 receptor expression (**C**,**F**) in the prefrontal cortex and hippocampus in adolescent (PND34) male and female rats. Data represent mean ± SEM (N = 5 rats/group). The concentration of proteins was calculated from a standard curve and expressed as ng/mg of protein. * *p* < 0.05; ** *p* < 0.01; *** *p* < 0.01 vs. Nonstressed.

Two-way ANOVA with repeated measures indicated the significant effect of MS (F(1,16) = 24.77; *p* < 0.001), but no sex effect (F(1,16) = 0.12; *p* > 0.05) and MS × sex interaction (F(1,16) = 0.05; *p* > 0.05) on DAT expression in the prefrontal cortex. Tukey’s post-hoc test showed that MS significantly increased DAT expression in the prefrontal cortex of males (*p* < 0.05) and females (*p* < 0.01), in comparison to nonseparated rats (Figure 3A). Two-way ANOVA with repeated measures indicated the significant effect of MS (F(1,16) = 8.33; *p* < 0.05) and MS × sex interaction (F(1,16) = 11.10; *p* < 0.01), but no sex effect (F(1,16) = 0.14; *p* > 0.05) on RIT2 expression in the prefrontal cortex. Tukey’s post-hoc test showed that MS significantly decreased RIT2 expression in the prefrontal cortex of females (*p* < 0.01) in comparison to nonseparated rats (Figure 3B). Two-way ANOVA with repeated measures indicated a significant effect of MS (F(1,16) = 28.10; *p* < 0.001), but no sex effect (F(1,16) = 0.91; *p* > 0.05) and MS × sex interaction (F(1,16) = 0.61; *p* > 0.05) on D1 expression in the prefrontal cortex. Tukey’s post-hoc test showed that MS significantly increased D1 expression in the prefrontal cortex of males (*p* < 0.05) and females (*p* < 0.01), in comparison to nonseparated rats (Figure 3C). Hence, MS induced upregulation of DAT and D1 receptor expression in the prefrontal cortex, with a greater effect in females. In addition, downregulation of RIT2 was observed only in females.

#### 2.3.2. Hippocampus

Two-way ANOVA with repeated measures indicated a significant effect of MS (F(1,16) = 41.79; *p* < 0.001), but no sex effect (F(1,16) = 4.21; *p* > 0.05) and MS × sex interaction (F(1,16) = 4.25; *p* > 0.05) on DAT expression in the hippocampus. Tukey’s post-hoc test showed that MS significantly increased DAT expression in the hippocampus of males (*p* < 0.05) and females (*p* < 0.001), in comparison to nonseparated rats (Figure 3D). Two-way ANOVA with repeated measures indicated a significant effect of MS (F(1,16) = 32.87; *p* < 0.001), but no sex effect (F(1,16) = 2.58; *p* > 0.05) and MS × sex interaction (F(1,16) = 2.81; *p* > 0.05) on RIT2 expression in the hippocampus. Tukey’s post-hoc test revealed that MS significantly decreased RIT2 expression in the prefrontal cortex of males (*p* < 0.05) and females (*p* < 0.001), in comparison to nonseparated rats (Figure 3E). Still, two-way ANOVA with repeated measures indicated a significant effect of MS (F(1,16) = 35.60; *p* < 0.001) and sex effect (F(1,16) = 9.13; *p* < 0.01), but no MS × sex interaction (F(1,16) = 0.23; *p* > 0.05) on D1 expression in the hippocampus. In addition, Tukey’s post-hoc test showed that MS significantly increased D1 expression in the hippocampus of males (*p* < 0.01) and females (*p* < 0.01) in comparison to nonseparated rats (Figure 3F). Generally, MS induced upregulation of all DAT and D1 receptor expression and downregulation of RIT2 expression, with much stronger effects in female rats, indicating that female rats were more vulnerable to MS.

### 2.4. The Influence of CE-123 Administration before Each Acquisition Session on D1 Receptor Expression Level in the Hippocampus of MS Male and Female Adolescent Rats

Two-way ANOVA with repeated measures indicated the significant effect of CE-123 at the dose of 10 mg/kg administered before each acquisition session (F(1,16) = 45.75; *p* < 0.001), but not sex factor (F(1,16) = 1.049; *p* > 0.05), on D1 receptor expression in the hippocampus of MS male and female rats. Interaction between these two factors was significant (F(1,16) = 6.427; *p* < 0.05). Tukey’s post-hoc test revealed that CE-123 administration decreased D1 receptor expression in both male (*p* < 0.05) and female (*p* < 0.001) (Table 1) MS rats, with a greater effect upon the females.

## 3. Discussion

Our results indicate that MS impairs the acquisition of spatial learning and memory in both male and female adolescent rats. The MS animals needed more time than the controls and made more errors in reaching the escape box during the acquisition phase of spatial learning in the Barnes maze task. These deleterious effects of MS on spatial learning and memory were more severe in female than male rats. CE-123, a DAT inhibitor, was found to improve learning in the MS rats. Notably, it was found to reduce latency and number of errors, with more evident effect in females. The proteomic analysis further supports the behavioral results, suggesting that MS upregulated DAT and dopamine D1 receptors expression in the prefrontal cortex and hippocampus. Regarding RIT2, the expression was decreased in the hippocampus for both the males and females, however, in the prefrontal cortex, reduction of expression was found only in the females, with no changes in the RIT2 expression in males. Overall, our data indicate that the deleterious effect of MS on learning and memory in adolescent rats may be due to hypofunction of dopamine signaling.

Evidence suggests that chronic early life stress has harmful effects on cognitive functions such as learning and memory in humans [7] and animals [53,54] in later life. Preclinical data reveal that, for example, MS [55] during the neonatal period (PND2-14, 180 min/day) reduces spatial memory formation in adult male rats (40 and 70 weeks old), indicating long term disruptive effects on brain structure and behavior. Another study shows that MS (PND2-5, 240 min/day, and then PND6-16, 480 min/day) with early weaning (PND17) slightly impairs working memory during non-stressful situations in female rats, but does not change spatial reference memory or associative learning under stressful circumstances in either sex [56]. In turn, our previous study demonstrated that MS (PND1-21, 180 min/day) induced deficits in the acquisition of spatial memory in the Barnes maze task in adolescent rats (PND30-33), with no significant differences between males and females [57]. However, in that study, data were pooled over 4 days of the memory development.

In the current study, when learning was analyzed each day of training, we found that the daily MS (PND1-21, 180 min/day) schedule indeed impaired spatial memory development in adolescent rats of both sexes (PND30-33), however, the female rats were more vulnerable to MS than males. Herein, the female MS rats (but not male MS rats) made significantly more errors and required longer time intervals to reach the escape box during the Day 2 acquisition phase than the controls. Published data confirmed that MS (24 h at PND3) affects hippocampal structural plasticity in adolescent (PND21) rats in a sex-dependent manner, and previous work demonstrated that neurogenesis is significantly increased in male, but decreased in female offspring after MS [58,59]. Accordingly, female rats (exposed to 24 h of MS at PND3) exhibited a lower total number of mature granule cells in adulthood [58], potently limiting the number of synaptic contacts that can be established in this region. Other research [60] indicates that the deleterious effects of MS on learning and memory are clearly age- and sex-dependent, but also rely on duration of the manipulation. However, we found that not only female, but also male rats showed deficits in learning procedure. In our study, a significant primary latency delay was observed in these animals on the 3rd and 4th day of the acquisition phase of the Barnes maze task. Such effects may suggest that male rats with MS experience exhibited significant impairment in retention of spatial information.

Studies in a variety of species have revealed that the development of the dopaminergic system is particularly sensitive towards prenatal stress [61,62] and neonatal stress experience [62,63,64,65,66,67]. Dopamine D1-like receptors in the hippocampus are especially noted for their critical role in spatial memory [27,28], and activating these receptors could enhance spatial learning and memory [25]. More recently, it has been reported that D1/D5 receptor signaling is associated with long-term memory consolidation [29] and working memory [30]. Thus, dopamine D1 receptor activation in the hippocampal regions can enhance memory and modulate memory consolidation or retrieval [31,32,33,68] through, for instance, modulation of excitatory amino acids (EAAs) release [68] and induction of long-term potentiation (LTP). 

Our present study showed that the expression of dopamine D1 receptors was upregulated in the hippocampus and prefrontal cortex of MS rats. Such effects were more pronounced in female than in male rats. Thus, our results suggest reduced dopamine transmission at the dopamine D1 receptors in adolescent rats induced by prolonged neonatal MS. Additionally, in previous work, the upregulation of D1 receptors has been observed in the hippocampus of adult rats after 15 min MS [4]. Other authors also have demonstrated that MS (24 h on PND9) increases the expression of dopamine D1 (and dopamine D2) receptors [69] in such brain structures as the prefrontal cortex and striatum in rats. However, further studies have indicated that chronic stress produces an increase in D1 receptor density in the prefrontal cortex, and drugs with dopamine-related actions improve spatial working memory in these subjects [22,70]. These findings suggest that an early life MS may alter the developmental trajectory of dopamine receptors, and this may have functional consequences on behavior in later life.

DAT is a membrane-bound presynaptic protein that rapidly clears dopamine that has been released into the extracellular space, and thereby limits the amplitude and duration of dopamine signaling. Previous studies have revealed reduced DAT expression in the midbrain and striatum of prenatally stressed mice [71] or upregulation of striatal DAT in rhesus monkeys [72]. Our study indicated upregulation of DAT in MS animals in the hippocampus and prefrontal cortex. This effect was most evident in the hippocampus of female rats. Our findings support a body of literature suggesting that prefrontal and hippocampal dopamine dysfunction might contribute to the cognitive and behavioral impairments reported in children from prenatally stressed pregnancies. An example of prenatal stress in children is attention deficit hyperactivity disorder (ADHD) [73]. Although results are mixed [74,75], ADHD patients are commonly treated with the DAT inhibitor, methylphenidate [76], although other DAT inhibitors, such as modafinil [77], were also clinically tested in ADHD. 

CE-123, as we previously stated, is a modafinil analog with increased affinity and selectivity for DAT [46,47]. In the current study, CE-123 (5 and 10 mg/kg) enhanced the acquisition of memory in the Barnes maze task in male and female adolescent rats (PND30-33) exposed to MS (PND1-21, 180 min/day) during the neonatal period. The effects of CE-123 were more pronounced in females than in males. The most effective dose was 10 mg/kg CE-123. This dose decreased latency and number of errors in both sexes of rats. Furthermore, CE-123 given during the memory acquisition phase had a beneficial effect on memory retention in the probe-trial test. The effects were more significant in females than in males. Together, our results support a functional link between dopaminergic neurotransmission and learning disabilities in rats with MS in the neonatal period, especially in females. 

DAT surface availability is dynamically regulated by endocytic trafficking, and direct PKC activation acutely diminishes DAT surface expression by accelerating DAT internalization. Previous cell line studies demonstrated that PKC-stimulated DAT endocytosis requires both Ack1 (a non-receptor tyrosine kinase) inactivation, which releases a DAT-specific endocytic brake, and the neuronal GTPase, RIT2, which binds DAT. However, it is unknown whether RIT2 is required for PKC-stimulated DAT endocytosis in dopaminergic terminals or whether there is region- and/or sex-dependent differences in PKC-stimulated DAT trafficking. Moreover, the mechanisms by which RIT2 controls PKC-stimulated DAT endocytosis are unknown [52].

Our results revealed that MS much more strongly decreased RIT2 expression in the hippocampus in female than in male rats. However, in the prefrontal cortex, MS decreased RIT2 only in the females, whereas RIT2 protein levels were unaffected in males. Thus, lack of changes in RIT2 expression in the prefrontal cortex in male rats can suggest sex and region-specific differences in DAT endocytic trafficking. Considering our outcomes, we hypothesize that the downregulation of RIT2 expression implies a lower control under PKC-stimulated DAT endocytosis in these brain structures.

However, our experiments have a limitation. Since DAT blockers can be considered as indirect dopamine receptor agonists and published data have shown that CE-123 administration in rats increased total DAT levels and D1 proteins levels in the synaptosomal fraction of hippocampal subregions CA1 and CA3 [47], we evaluated only the impact of CE-123 on upregulation of D1 receptors (but not DAT) in the hippocampus of MS rats. Herein, our results showed that CE-123 decreased the D1 receptor upregulation, as we revealed in Table 1. Furthermore, although published data indicated close relationships between RIT2 and D1 receptors in male striatal function, as well as dopamine-dependent behavior [78], we did not assess the influence of CE-123 on RIT2 expression. Thus, our data only partially supports the parallelism between the behavior and the biomarkers. This subject will be addressed in a future study.

Collectively, our experiments indicate that MS induced deficits in the acquisition of learning and memory in the Barnes maze test in both groups of adolescent rats: male and female. The effects were more pronounced in female than in male rats. Thus, we suggest that females are more vulnerable to cognitive impairments that are induced by mother neglect during the neonatal period. Moreover, neonatal MS induces hypofunction of dopaminergic transmission in adolescent rats (with DAT overexpression and upregulation of D1 receptors) that is decreased by CE-123, an atypical DAT inhibitor (as indicated in our preliminary study in the hippocampus), leading to amelioration of the spatial memory impairment in MS rats. Hence, CE-123 administration could inhibit DAT upregulation, normalize dopamine signaling at the D1 receptor, and effectively improve learning and memory in MS rats. This is the first study that reveals the potential usefulness of DAT inhibitors, such as CE-123, in restoring spatial memory deficits induced by MS. However, more research is needed.

## 4. Materials and Methods

### 4.1. Animals

The study was approved by the Local Ethics Committee (05/2022) in Lublin under the ‘3R approach’ (Replace, Reduce and Refine) and performed according to the National Institute of Health Guidelines for the Care and Use of Laboratory Animals, as well as the European Community Council Directive of November 2010 for Care and Use of Laboratory Animals (Directive, 2010/63/EU) (IACUC equivalent approval). The subjects used in the present study were offspring of Wistar dams (OMD, Lublin, Poland) mated in the animal facility at the OMD, Lublin, Poland. The dams were housed individually throughout the gestation in polypropylene cages (41 × 34 × 16 cm) with approximately 3 cm layer of sawdust shavings on the cage floor. Rodent chow (Sniff Specialization GmbH, Sorest, Germany) and water were available ad libitum to the animals throughout the study. The experiments were maintained under standard laboratory conditions (22 ± 1 °C, 12:12 light/dark cycle, lights on at 8:00). All behavioral experiments were performed between 9:00 a.m. and 7:00 p.m. The day of birth was designated as PND 0.

### 4.2. Drugs

S-CE-123 (5-((benzhydrylsulfinyl)methyl) thiazole) (CE-123), a modafinil analog, was synthesized in the Lubec laboratory (University of Vienna, Vienna, Austria) as reported previously [46]. On the day of the experiments, CE-123 was freshly dissolved in a vehicle of 1% DMSO and 3.3% Tween 80 diluted in 0.9% NaCl. CE-123 was administered intraperitoneally (i.p.) at the doses of 5 or 10 mg/kg in a value of 1 mL/kg of body weight 30 min prior to each acquisition session. The CE-123 doses were selected based on previous research [46,47,48]. Controls were administered by vehicle alone.

### 4.3. Maternal Separation

The maternal separation (MS) procedure was carried out from PND1 to PND21. The total number of dams used in the present study was 20 (10 litters for MS stress and 10 litters for control). The procedure of MS was based on the protocol described by Chocyk et al. [79] with minor modifications. On each of PND1–21, the dams and the pups were removed from the maternity cages for 180 min (09:00 to 12:00). The mothers were placed individually in holding cages, while each litter was placed in a cardboard container lined with fresh bedding material. The containers were moved to a bigger cage where adequate ambient temperature (34 °C) was provided by placing hot water bottles on the bottom. After the 180 min separation, the pups and the dams were returned to the maternity cages. Once a week, the maternity cages were cleaned during one of the separation procedures. Control, non-separated (NS) animals were left undisturbed with their mothers, except during the cage cleaning that was performed once a week. During the MS procedure, male and female segregation was not performed. After weaning, offspring at PND 22 were separated according to sex, housed 5 per cage, and assigned to the Barnes maze task (PND29). Male and female offspring were used in the present study (N = 10 rats/group).

### 4.4. Barnes Maze Task

The Barnes maze task was carried out according to the method described in our earlier study [80]. The Barnes maze apparatus (Stoelting, Dublin, Ireland) consisted of a circular grey metal platform (diameter 122 cm), elevated 100 cm above the floor, with 20 holes (10 cm diameter) located in its periphery. One hole was connected to an escape box of 35 cm × 12 cm × 12 cm of the same material and color as the platform. The other holes were covered underneath with a flat box, also of the same material and color, so that the rats could not discriminate the escape hole from other holes until situated adjacent to it. In addition, numerous visual cues (in the form of large colorful geometric shapes) were placed on the walls of the testing room at 1–2 m distance from the edge of the maze. To evoke the potentiated escape response, the platform was brightly lit (two points of light 1.5 m above the maze: 500 W each) as an aversive stimulus. The Barnes maze task was run in two phases: habituation and acquisition trial.

#### 4.4.1. Habituation

On PND29, one day before the acquisition phase, to reduce anxiety behavior, the rats were habituated to the platform and the escape box. This habituation trial was performed with the lights on.

#### 4.4.2. Acquisition Phase

One day (24 h) after the maze habituation, the same rats were subjected to the acquisition phase (PND30-33). Acquisition involved one training session per day for 4 consecutive days. Each training session consisted of two 180 s trials, with a 5-min inter-trial interval when the animals were returned to their home cages. CE-123 at the doses of 5 or 10 mg/kg was administered once daily 30 min before every first acquisition trial for 4 days of the acquisition phase. The location of the platform and the escape box remained constant over all the acquisition trials. Each trial began by placing the animal at the center of the platform and then it was allowed to freely explore the apparatus. The trial was completed after 180 s or when the animal entered the escape box. Immediately after entering the escape box, the hole was covered for 30 s before the rat was returned to its home cage. If the animal did not enter the goal box within 180 s, it was gently guided there by the experimenter and could explore it for 30 s. To dissipate odor cues and to provide a standard olfactory context for each trial, the platform surface and the escape box were wiped with a 10% (*w*/*v*) ethanol solution after each trial [80,81]. All trials were recorded by a trained observer. Since the animals occasionally lacked motivation and merely explored the maze after finding the escape box rather than entering it, following the work of many authors [82,83,84,85], in our experiments, we scored such parameters as the primary latency and primary errors. Primary latency was defined as the time required for the rat to make initial contact with the escape box. Primary errors were defined as the number of holes visited before the first contact with the escape box.

#### 4.4.3. Probe-Trial

To evaluate spatial memory retrieval, 24 h after the acquisition phase, the subjects (PND34) received a probe-trial for 180 s. During this trial, the rats were allowed to explore the maze. The primary latency and primary errors to reach the escape box were counted. On the day of the probe trial, no substances were administered to the animals.

### 4.5. ELISA Assays

Quantitative measurement (PND33) of dopamine receptor 1 (D1), dopamine transporter (DAT) and GTP-binding protein RIT2 in such brain structures as the prefrontal cortex and hippocampus was performed using a Rat D1 receptor ELISA kit (Reddot Biotech, Kelowna, BC, Canada), a Rat Dopamine Transporter (DAT) ELISA kit (E0222Ra; Bioassay Technology Laboratory, Shanghai, China) and Rat RIT2 (GTP-Binding Protein Rit2) ELISA Kit (ELK9277; ELK Biotechnology, Wuhan, China), respectively, following manufacturers’ protocols. Firstly, frozen rat brain structures were homogenized in ice-cold PBS pH 7.4 containing cocktails of protease and phosphatase inhibitors (Sigma-Aldrich, Saint Louis, MO, USA) using a homogenizer ball (Bioprep-24, Aosheng, Hangzhou, China) (10 s at 10,000 rpm). Then, homogenates were centrifuged for 5 min at 5000× *g* and the supernatants were immediately removed. Duplicates of each sample and series of standards were transferred to ELISA plates. The absorbance was measured at a wavelength of λ = 450 nm using a Multiskan Spectrum spectrophotometer (Thermo LabSystems, Philadelphia, PA, USA). The concentration of proteins was calculated from a standard curve and expressed as ng/mg of protein. For protein measurement, the bicinchoninic acid assay (BCA) protein assay kit (Serva, Heidelberg, Germany) was used. ELISA assays were performed after completion of the Barnes maze task in the vehicle- and CE-123-treated animals.

### 4.6. Statistical Analysis

The obtained results were analyzed using Prism v. 8.0.0 for Windows (GraphPad Software, San Diego, CA, USA). The statistical significance of drug effects from behavioral and biochemical tests was analyzed by the two-way analysis of variance (ANOVA) with repeated measures. This was followed by Tukey’s post-hoc test. The results were presented as means ± standard errors of means (SEM) of values. *p* value less than 0.05 was considered statistically significant for all tests.

## Figures and Tables

**Table 1:** Effect of CE-123 administration (10 mg/kg) before each acquisition session on D1 receptor expression in the hippocampus of MS male and female adolescent (PND34) rats. Data represent mean ± SEM (N = 5 rats/group). The concentration of proteins was calculated from a standard curve and expressed as ng/mg of protein. * *p* < 0.05; *** *p*< 0.001 vs. stressed rats.

FEMALE		MALE	
STRESSED	STRESSED + CE 10 mg	STRESSED	STRESSED + CE 10 mg
230.611 ± 6.967	162.233 ± 2.354 ***	219.500 ± 4.962	188.406 ± 8.308 *

## Data Availability

The data presented in this study are available on request from the corresponding author.

## References

[1] Lupien S.J., McEwen B.S., Gunnar M.R., Heim C. (2009). Effects of stress throughout the lifespan on the brain, behaviour and cognition. Nat. Rev. Neurosci..

[2] Huot R.L., Plotsky P.M., Lenox R.H., McNamara R.K. (2002). Neonatal maternal separation reduces hippocampal mossy fiber density in adult Long Evans rats. Brain Res..

[3] Matthews K., Robbins T.W. (2003). Early experience as a determinant of adult behavioural responses to reward: The effects of repeated maternal separation in the rat. Neurosci. Biobehav. Rev..

[4] Ploj K., Roman E., Nylander I. (2003). Long-term effects of short and long periods of maternal separation on brain opioid peptide levels in male Wistar rats. Neuropeptides.

[5] Pachenari N., Azizi H., Ghasemi E., Azadi M., Semnanian S. (2018). Exposure to opiates in male adolescent rats alters pain perception in the male offspring. Behav. Pharmacol..

[6] Goodman J.B., Freeman E.E., Chalmers K.A. (2019). The relationship between early life stress and working memory in adulthood: A systematic review and meta-analysis. Memory.

[7] Pechtel P., Pizzagalli D.A. (2011). Effects of early life stress on cognitive and affective function: An integrated review of human literature. Psychopharmacology.

[8] Paterniti S., Sterner I., Caldwell C., Bisserbe J.C. (2017). Childhood neglect predicts the course of major depression in a tertiary care sample: A follow-up study. BMC Psychiatry.

[9] Wigger A., Neumann I.D. (1999). Periodic maternal deprivation induces gender-dependent alterations in behavioral and neuroendocrine responses to emotional stress in adult rats. Physiol. Behav..

[10] Kalinichev M., Easterling K.W., Plotsky P.M., Holtzman S.G. (2002). Long-lasting changes in stress-induced corticosterone response and anxiety-like behaviors as a consequence of neonatal maternal separation in Long-Evans rats. Pharmacol. Biochem. Behav..

[11] Muhammad A., Kolb B. (2011). Maternal separation altered behavior and neuronal spine density without influencing amphetamine sensitization. Behav. Brain Res..

[12] Tsuda M.C., Ogawa S. (2012). Long-lasting consequences of neonatal maternal separation on social behaviors in ovariectomized female mice. PLoS ONE.

[13] Niwa M., Matsumoto Y., Mouri A., Ozaki N., Nabeshima T. (2011). Vulnerability in early life to changes in the rearing environment plays a crucial role in the aetiopathology of psychiatric disorders. Int. J. Neuropsychopharmacol..

[14] Thomas A.W., Caporale N., Wu C., Wilbrecht L. (2016). Early maternal separation impacts cognitive flexibility at the age of first independence in mice. Dev. Cogn. Neurosci..

[15] Chiba A.A., Kesner R.P., Reynolds A.M. (1994). Memory for spatial location as a function of temporal lag in rats: Role of hippocampus and medial prefrontal cortex. Behav. Neural. Biol..

[16] Morris R.G., Garrud P., Rawlins J.N., O’Keefe J. (1982). Place navigation impaired in rats with hippocampal lesions. Nature.

[17] Fuge P., Aust S., Fan Y., Weigand A., Gärtner M., Feeser M., Bajbouj M., Grimm S. (2014). Interaction of early life stress and corticotropin-releasing hormone receptor gene: Effects on working memory. Biol. Psychiatry.

[18] O’Keefe J. (1999). Do hippocampal pyramidal cells signal non-spatial as well as spatial information?. Hippocampus.

[19] Muzzio I.A., Kentros C., Kandel E. (2009). What is remembered? Role of attention on the encoding and retrieval of hippocampal representations. J. Physiol..

[20] Kentros C.G., Agnihotri N.T., Streater S., Hawkins R.D., Kandel E.R. (2004). Increased attention to spatial context increases both place field stability and spatial memory. Neuron.

[21] Gasbarri A., Sulli A., Innocenzi R., Pacitti C., Brioni J.D. (1996). Spatial memory impairment induced by lesion of the mesohippocampal dopaminergic system in the rat. Neuroscience.

[22] Mizoguchi K., Yuzurihara M., Ishige A., Sasaki H., Chui D.H., Tabira T. (2000). Chronic stress induces impairment of spatial working memory because of prefrontal dopaminergic dysfunction. J. Neurosci..

[23] Broussard J.I., Yang K., Levine A.T., Tsetsenis T., Jenson D., Cao F., Garcia I., Arenkiel B.R., Zhou F.M., De Biasi M. (2016). Dopamine regulates aversive contextual learning and associated in vivo synaptic plasticity in the hippocampus. Cell Rep..

[24] Hansen N., Manahan-Vaughan D. (2014). Dopamine D1/D5 receptors mediate informational saliency that promotes persistent hippocampal long-term plasticity. Cereb. Cortex..

[25] Kempadoo K.A., Mosharov E.V., Choi S.J., Sulzer D., Kandel E.R. (2016). Dopamine release from the locus coeruleus to the dorsal hippocampus promotes spatial learning and memory. Proc. Natl. Acad. Sci. USA.

[26] El-Ghundi M., O’Dowd B.F., George S.R. (2007). Insights into the role of dopamine receptor systems in learning and memory. Rev. Neurosci..

[27] da Silva W.C., Köhler C.C., Radiske A., Cammarota M. (2012). D1/D5 dopamine receptors modulate spatial memory formation. Neurobiol. Learn Mem..

[28] Xing B., Kong H., Meng X., Wei S.G., Xu M., Li S.B. (2010). Dopamine D1 but not D3 receptor is critical for spatial learning and related signaling in the hippocampus. Neuroscience.

[29] Karunakaran S., Chowdhury A., Donato F., Quairiaux C., Michel C.M., Caroni P. (2016). PV plasticity sustained through D1/5 dopamine signaling required for long-term memory consolidation. Nat. Neurosci..

[30] Williams G.V., Castner S.A. (2006). Under the curve: Critical issues for elucidating D1 receptor function in working memory. Neuroscience.

[31] Balderas I., Moreno-Castilla P., Bermudez-Rattoni F. (2013). Dopamine D1 receptor activity modulates object recognition memory consolidation in the perirhinal cortex but not in the hippocampus. Hippocampus.

[32] Hotte M., Thuault S., Lachaise F., Dineley K.T., Hemmings H.C., Nairn A.C., Jay T.M. (2006). D1 receptor modulation of memory retrieval performance is associated with changes in pCREB and pDARPP-32 in rat prefrontal cortex. Behav. Brain Res..

[33] Rossato J.I., Radiske A., Kohler C.A., Gonzalez C., Bevilaqua L.R., Medina J.H., Cammarota M. (2013). Consolidation of object recognition memory requires simultaneous activation of dopamine D1/D5 receptors in the amygdala and medial prefrontal cortex but not in the hippocampus. Neurobiol. Learn Mem..

[34] Kristensen A.S., Andersen J., Jørgensen T.N., Sørensen L., Eriksen J., Loland C.J., Strømgaard K., Gether U. (2011). SLC6 neurotransmitter transporters: Structure, function, and regulation. Pharmacol. Rev..

[35] Gainetdinov R.R., Jones S.R., Fumagalli F., Wightman R.M., Caron M.G. (1998). Re-evaluation of the role of the dopamine transporter in dopamine system homeostasis. Brain Res. Brain Res. Rev..

[36] Kurzina N.P., Aristova I.Y., Volnova A.B., Gainetdinov R.R. (2020). Deficit in working memory and abnormal behavioral tactics in dopamine transporter knockout rats during training in the 8-arm maze. Behav. Brain Res..

[37] Efimova E.V., Gainetdinov R.R., Budygin E.A., Sotnikova T.D. (2016). Dopamine transporter mutant animals: A translational perspective. J. Neurogenet..

[38] Battleday R.M., Brem A.K. (2015). Modafinil for cognitive neuroenhancement in healthy non-sleep-deprived subjects: A systematic review. Eur. Neuropsychopharmacol..

[39] Bobo W.V., Woodward N.D., Sim M.Y., Jayathilake K., Meltzer H.Y. (2011). The effect of adjunctive armodafinil on cognitive performance and psychopathology in antipsychotic-treated patients with schizophrenia/schizoaffective disorder: A randomized, double-blind, placebo-controlled trial. Schizophr. Res..

[40] Dolder P.C., Müller F., Schmid Y., Borgwardt S.J., Liechti M.E. (2018). Direct comparison of the acute subjective, emotional, autonomic, and endocrine effects of MDMA, methylphenidate, and modafinil in healthy subjects. Psychopharmacology.

[41] Alam N., Choudhary K. (2018). Haloperidol attenuates methylphenidate and modafinil induced behavioural sensitization and cognitive enhancement. Metab. Brain Dis..

[42] Robinson T.E., Browman K.E., Crombag H.S., Badiani A. (1998). Modulation of the induction or expression of psychostimulant sensitization by the circumstances surrounding drug administration. Neurosci. Biobehav. Rev..

[43] Gerrard P., Malcolm R. (2007). Mechanisms of modafinil: A review of current research. Neuropsychiatr. Dis. Treat..

[44] Kalaba P., Aher N.Y., Ilić M., Dragačević V., Wieder M., Miklosi A.G., Zehl M., Wackerlig J., Roller A., Beryozkina T. (2017). Heterocyclic analogues of modafinil as novel, atypical dopamine transporter inhibitors. J. Med. Chem..

[45] Kalaba P., Ilić M., Aher N.Y., Dragačević V., Wieder M., Zehl M., Wackerlig J., Beyl S., Sartori S.B., Ebner K. (2020). Structure-activity relationships of novel thiazole-based modafinil analogues acting at monoamine transporters. J. Med. Chem..

[46] Nikiforuk A., Kalaba P., Ilic M., Korz V., Dragačević V., Wackerlig J., Langer T., Höger H., Golebiowska J., Popik P. (2017). A novel dopamine transporter inhibitor CE-123 improves cognitive flexibility and maintains impulsivity in healthy male rats. Front. Behav. Neurosci..

[47] Kristofova M., Aher Y.D., Ilic M., Radoman B., Kalaba P., Dragacevic V., Aher N.Y., Leban J., Korz V., Zanon L. (2018). A daily single dose of a novel modafinil analogue CE-123 improves memory acquisition and memory retrieval. Behav. Brain Res..

[48] Gibula-Tarlowska E., Korz V., Lopatynska-Mazurek M., Chlopas-Konowalek A., Grochecki P., Kalaba P., Dragacevic V., Kotlinski R., Kujawski R., Szulc M. (2021). CE-123, a novel dopamine transporter inhibitor, attenuates locomotor hyperactivity and improves cognitive functions in rat model of fetal alcohol spectrum disorders. Behav. Brain Res..

[49] Sagheddu C., Pintori N., Kalaba P., Dragačević V., Piras G., Lubec J., Simola N., De Luca M.A., Lubec G., Pistis M. (2020). Neurophysiological and neurochemical effects of the putative cognitive enhancer (*S*)-CE-123 on mesocorticolimbic dopamine system. Biomolecules.

[50] Lee C.H., Della N.G., Chew C.E., Zack D.J. (1996). Rin, a neuron-specific and calmodulin-binding small G-protein, and Rit define a novel subfamily of ras proteins. J. Neurosci..

[51] Navaroli D.M., Stevens Z.H., Uzelac Z., Gabriel L., King M.J., Lifshitz L.M., Sitte H.H., Melikian H.E. (2011). The plasma membrane-associated GTPase Rin interacts with the dopamine transporter and is required for protein kinase C-regulated dopamine transporter trafficking. J. Neurosci..

[52] Fagan R.R., Kearney P.J., Sweeney C.G., Luethi D., Schoot Uiterkamp F.E., Schicker K., Alejandro B.S., O’Connor L.C., Sitte H.H., Melikian H.E. (2020). Dopamine transporter trafficking and Rit2 GTPase: Mechanism of action and in vivo impact. J. Biol. Chem..

[53] Judo C., Matsumoto M., Yamazaki D., Hiraide S., Yanagawa Y., Kimura S., Shimamura K., Togashi H. (2010). Early stress exposure impairs synaptic potentiation in the rat medial prefrontal cortex underlying contextual fear extinction. Neuroscience.

[54] Sandi C., Pinelo-Nava M.T. (2007). Stress and memory: Behavioral effects and neurobiological mechanisms. Neural. Plast..

[55] Sousa V.C., Vital J., Costenla A.R., Batalha V.L., Sebastião A.M., Ribeiro J.A., Lopes L.V. (2014). Maternal separation impairs long term-potentiation in CA1-CA3 synapses and hippocampal-dependent memory in old rats. Neurobiol. Aging..

[56] Sun X., Zhang Y., Li X., Liu X., Qin C. (2021). Early-life neglect alters emotional and cognitive behavior in a sex-dependent manner and reduces glutamatergic neuronal excitability in the prefrontal cortex. Front Psychiatry.

[57] Filarowska-Jurko J., Komsta L., Smaga I., Surowka P., Marszalek-Grabska M., Grochecki P., Nizio D., Filip M., Kotlinska J.H. (2022). Maternal separation alters ethanol drinking and reversal learning processes in adolescent rats: The impact of sex and glycine transporter type 1 (GlyT1) inhibitor. Int. J. Mol. Sci..

[58] Oomen C.A., Girardi C.E., Cahyadi R., Verbeek E.C., Krugers H., Joëls M., Lucassen P.J. (2009). Opposite effects of early maternal deprivation on neurogenesis in male versus female rats. PLoS ONE.

[59] Loi M., Koricka S., Lucassen P.J., Joëls M. (2014). Age- and sex-dependent effects of early life stress on hippocampal neurogenesis. Front. Endocrinol..

[60] Kosten T.A., Kim J.J., Lee H.J. (2012). Early life manipulations alter learning and memory in rats. Neurosci. Biobehav. Rev..

[61] Braun K., Bock J., Wainstock T., Matas E., Gaisler-Salomon I., Fegert J., Ziegenhain U., Segal M. (2020). Experience-induced transgenerational (re-)programming of neuronal structure and functions: Impact of stress prior and during pregnancy. Neurosci. Biobehav. Rev..

[62] Bock J., Wainstock T., Braun K., Segal M. (2015). Stress in utero: Prenatal programming of brain plasticity and cognition. Biol. Psychiatry.

[63] Matas E., Bock J., Braun K. (2016). The impact of parent-infant interaction on epigenetic plasticity mediating synaptic adaptations in the infant brain. Psychopathology.

[64] Kunzler J., Braun K., Bock J. (2015). Early life stress and sex-specific sensitivity of the catecholaminergic systems in prefrontal and limbic regions of Octodon degus. Brain Struct. Funct..

[65] Ziabreva I., Schnabel R., Poeggel G., Braun K. (2003). Mother’s voice “buffers” separation-induced receptor changes in the prefrontal cortex of octodon degus. Neuroscience.

[66] Kasanova Z., Hernaus D., Vaessen T., van Amelsvoort T., Winz O., Heinzel A., Pruessner J., Mottaghy F.M., Collip D., Myin-Germeys I. (2016). Early-life stress affects stress-related prefrontal dopamine activity in healthy adults, but not in individuals with psychotic disorder. PLoS ONE.

[67] Majcher-Maślanka I., Solarz A., Wędzony K., Chocyk A. (2017). The effects of early-life stress on dopamine system function in adolescent female rats. Int. J. Dev. Neurosci..

[68] Wang M., Datta D., Enwright J., Galvin V., Yang S.T., Paspalas C., Kozak R., Gray D.L., Lewis D.A., Arnsten A.F.T. (2019). A novel dopamine D1 receptor agonist excites delay-dependent working memory-related neuronal firing in primate dorsolateral prefrontal cortex. Neuropharmacology.

[69] Rentesi G., Antoniou K., Marselos M., Syrrou M., Papadopoulou-Daifoti Z., Konstandi M. (2013). Early maternal deprivation-induced modifications in the neurobiological, neurochemical and behavioral profile of adult rats. Behav. Brain Res..

[70] Banqueri M., Gutiérrez-Menéndez A., Méndez M., Conejo N.M., Arias J.L. (2021). Early life stress due to repeated maternal separation alters the working memory acquisition brain functional network. Stress.

[71] Son G.H., Chung S., Geum D., Kang S.S., Choi W.S., Kim K., Choi S. (2007). Hyperactivity and alteration of the midbrain dopaminergic system in maternally stressed male mice offspring. Biochem. Biophys. Res. Commun..

[72] Converse A.K., Moore C.F., Moirano J.M., Ahlers E.O., Larson J.A., Engle J.W., Barnhart T.E., Murali D., Christian B.T., DeJesus O.T. (2013). Prenatal stress induces increased striatal dopamine transporter binding in adult nonhuman primates. Biol. Psychiatry.

[73] Van den Bergh B.R., Marcoen A. (2004). High antenatal maternal anxiety is related to ADHD symptoms, externalizing problems, and anxiety in 8- and 9-year-olds. Child Dev..

[74] Spencer T.J., Biederman J., Mick E. (2007). Attention-deficit/hyperactivity disorder: Diagnosis, lifespan, comorbidities, and neurobiology. J. Pediatr. Psychol..

[75] Volkow N.D., Wang G.J., Kollins S.H., Wigal T.L., Newcorn J.H., Telang F., Fowler J.S., Zhu W., Logan J., Ma Y. (2009). Evaluating dopamine reward pathway in ADHD: Clinical implications. JAMA.

[76] Bannon M.J. (2005). The dopamine transporter: Role in neurotoxicity and human disease. Toxicol. Appl. Pharmacol..

[77] Turner D. (2006). A review of the use of modafinil for attention-deficit hyperactivity disorder. Expert Rev. Neurother..

[78] Sweeney C.G., Kearney P.J., Fagan R.R., Smith L.A., Bolden N.C., Zhao-Shea R., Rivera I.V., Kolpakova J., Xie J., Gao G. (2020). Conditional, inducible gene silencing in dopamine neurons reveals a sex-specific role for Rit2 GTPase in acute cocaine response and striatal function. Neuropsychopharmacology.

[79] Chocyk A., Dudys D., Przyborowska A., Maćkowiak M., Wędzony K. (2010). Impact of maternal separation on neural cell adhesion molecules expression in dopaminergic brain regions of juvenile, adolescent and adult rats. Pharmacol. Rep..

[80] Grochecki P., Smaga I., Lopatynska-Mazurek M., Gibula-Tarlowska E., Kedzierska E., Listos J., Talarek S., Marszalek-Grabska M., Hubalewska-Mazgaj M., Korga-Plewko A. (2021). Effects of mephedrone and amphetamine exposure during adolescence on spatial memory in adulthood: Behavioral and neurochemical analysis. Int. J. Mol. Sci..

[81] Lopatynska-Mazurek M., Antolak A., Grochecki P., Gibula-Tarlowska E., Bodzon-Kulakowska A., Listos J., Kedzierska E., Suder P., Silberring J., Kotlinska J.H. (2021). Rapamycin improves spatial learning deficits, vulnerability to alcohol addiction and altered expression of the GluN2B subunit of the NMDA receptor in adult rats exposed to ethanol during the neonatal period. Biomolecules.

[82] Harrison F.E., Reiserer R.S., Tomarken A.J., McDonald M.P. (2006). Spatial and nonspatial escape strategies in the Barnes maze. Learn Mem..

[83] Li L., Csaszar E., Szodorai E., Patil S., Pollak A., Lubec G. (2014). The differential hippocampal phosphoproteome of Apodemus sylvaticus paralleling spatial memory retrieval in the Barnes maze. Behav. Brain Res..

[84] Marszalek-Grabska M., Gibula-Bruzda E., Bodzon-Kulakowska A., Suder P., Gawel K., Talarek S., Listos J., Kedzierska E., Danysz W., Kotlinska J.H. (2018). ADX-47273, a mGlu5 receptor positive allosteric modulator, attenuates deficits in cognitive flexibility induced by withdrawal from ‘binge-like’ ethanol exposure in rats. Behav. Brain Res..

[85] Gawel K., Gibula E., Marszalek-Grabska M., Filarowska J., Kotlinska J.H. (2019). Assessment of spatial learning and memory in the Barnes maze task in rodents-methodological consideration. Naunyn Schmiedebergs Arch. Pharmacol..

